# Marrying oral tribology to sensory perception: a systematic review

**DOI:** 10.1016/j.cofs.2019.05.007

**Published:** 2019-06

**Authors:** Anwesha Sarkar, Emma M Krop

**Affiliations:** Food Colloids and Bioprocessing Group, School of Food Science and Nutrition, University of Leeds, LS2 9JT, Leeds, UK

## Abstract

Oral tribology is rapidly entering into the food scientists’ toolbox because of its promises to predict surface-related mouthfeel perception. In this systematic review, we discuss how oral tribology relates to specific sensory attributes in model and real foods focussing on recent literature from 2016 onwards. Electronic searches were conducted in four databases, yielding 4857 articles which were narrowed down to a set of 16 articles using pre-specified criteria. New empirical correlations have emerged between friction coefficients in the mixed lubrication regime and fat-related perception (e.g. smoothness) as well as non-fat-related perception (e.g. pastiness, astringency, stickiness). To develop mechanistically supported generalized relationships, we recommend coupling tribological surfaces and testing conditions that are harmonized across laboratories with temporal sensory testing and multivariate statistical analysis.

## Highlights

• Systematic review was conducted on relating oral tribology to sensory perception.
• Friction coefficient (*μ*) is measured using various surfaces and testing conditions.
• Both model and real foods have shown friction-sensory relations across laboratories.
• Empirical relations exist between *μ* and sensory attributes (astringent, smooth).
• Harmonized tribology testing condition is key to develop generalized correlations.

## Graphical abstract

**Figure:**
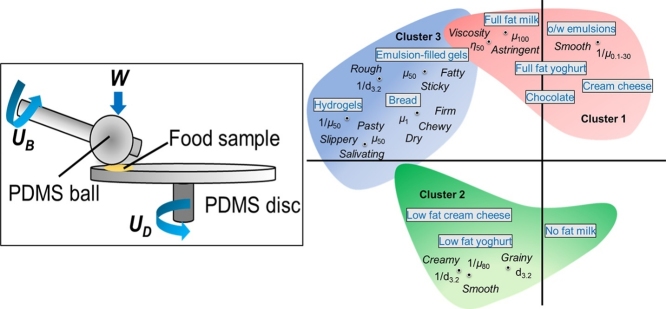

**Current Opinion in Food Science** 2019, **27**:64–73

This review comes from a themed issue on **Food physics & materials science**

Edited by **Elke Scholten**

For a complete overview see the Issue and the Editorial

Available online 2nd June 2019

**https://doi.org/10.1016/j.cofs.2019.05.007**

2214-7993/© 2019 The Authors. Published by Elsevier Ltd. This is an open access article under the CC BY license (http://creativecommons.org/licenses/by/4.0/).

## Introduction

Tribology, that is, the science of friction, lubrication and wear in interacting surfaces in relative motion, is no longer limited to answering mechanical engineering research questions. Specifically, ‘oral tribology’ associated with understanding the interaction of food with saliva-coated oral surfaces (tongue, palate, teeth, mucosa) is gaining a greater momentum in food oral processing research. Soft tribology to measure friction coefficients (*μ*) in presence of model food structures, such as aqueous hydrogels [1^••^], emulsions [2^•^], emulsion gels [3], microgels [4,5] as well as real food products [6^•^,^7^, ^8^, ^9^, ^10^] is emerging as a quantitative tool in various food physics laboratories across the globe. This might be attributed to the correlations recently observed between instrumental *μ* values at particular speeds and certain sensory attributes that would not have been assessable with conventional bulk rheological measurements alone [11^••^]. However, a number of challenges on testing procedures as well as underlying mechanisms behind such correlations remain unresolved.

Considering the fast-moving nature of this research field, we present the first *systematic review* on the relationships between instrumental tribology measurements and sensory perception. We discuss here only the recent studies involving model and real food systems from 2016 onwards. We conclude by summarizing a list of challenges and opportunities on the road to identify generalized relationships between *μ* and specific sensory attributes. To obtain a fundamental understanding of ‘oral tribology’, the underlying theoretical principles and how the polymeric surfaces used in *in vitro* tribological set-ups and *ex vivo* set-ups mimic/differ from real mouth surfaces, we recommend our recent review on soft lubrication [11^••^]. We also recommend previous articles of importance in oral tribology and oral processing [12,13^•^] and specifically papers that reviewed the oral tribology–sensory relationships in literature published before 2016 [14^•^,15^•^]. On the basis of these reviews, some of the key developments and research challenges addressed in oral tribology research until 2016 have been:

- Direct employment of various commercial tribometers and conditions used in the mechanical engineering discipline for conducting tribological measurements of food samples, such as milks, yoghurts, custards;
- Development of bespoke tribometers in different labs e.g. optical tribometer cell (OTC) for visualising microstructure while tribo-shearing, adaptation of texture analyser for friction force measurement;
- Use of animal tissues as tribological surfaces in addition to steel surfaces;
- Relating the friction coefficient to sensory attributes, focussing mainly on ‘astringency’ and to some extent on ‘creaminess’;
- Fitting the friction coefficient versus entrainment speed data to a Master curve, latter containing the rheological component.

Although oral tribology has started to herald remarkable applications in other fields of food science, such as detection of adulteration in foods [16,17] and promoting satiation [18,19], such studies fall outside of the scope of this review.

## Methodology

The 2009 PRISMA (Preferred Reporting Items for Systematic Reviews and Meta-Analysis) guidelines were used for reporting this systematic review. A comprehensive literature search was conducted using four online databases: Science Direct (Elsevier), Web of Science (Clarivate Analytics), Scopus (Elsevier) and American Chemical Society (ACS) publications. The searches were conducted on 4 and 5 February 2019. Only articles published in English were included and no time limit was set initially. The objective was to find all studies linking instrumental tribology data to sensory measurements of any type of food (both model and real foods). A broad range of search terms were used to increase the chance of locating all relevant literature. We used a combination of relevant keywords including tribology, tribometer, lubrication, friction, Stribeck, sensory, perception, taste, after-taste, cream* and astringen*. The search strategy was validated by checking that a number articles familiar by both the authors were indeed retrieved in at least one of the databases. The citations of all articles were exported to the reference software Endnote X8 for further analysis.

**Figure 1:**
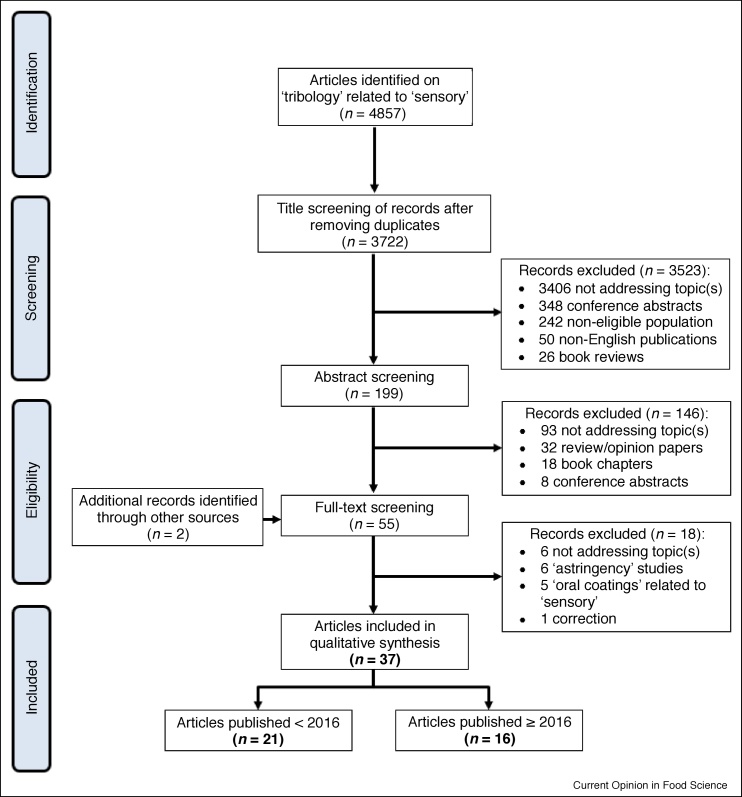
PRISMA flow-chart of the study selection procedure for qualitative synthesis of articles in the tribology–sensory area published in 2016 and onwards.

Only original research reports of human studies were included in this systematic review. A summary of the study selection procedure (PRISMA four-phase flow diagram) is shown in Figure 1. A total of 4857 articles were initially identified; after that the number was reduced to 3722 after removing any duplicates.

After the title screening, a further 3523 articles were excluded for various reasons. Some did not address the topic of interest (3406), focussed on a non-eligible population such as infants, older adults or participants with certain diseases (242), consisted of a conference abstract (348) or book review (26), or were not written in English (50). Some articles were excluded for multiple of these reasons; therefore, the total number of articles is lower than the sum. The remaining 199 articles were screened for their abstract, and a further 146 articles were excluded: an additional 93 articles did not properly address the topic, 32 were review/opinion papers and 18 book chapters without original research, and 8 were conference abstracts. Two additional articles were identified by scanning reference lists from other publications.

Finally, after assessing the full-text of the remaining 55 articles, 37 potentially relevant studies were identified. Six more articles were excluded that did not consider the topic of interest (three studies did not include sensory texture measurements, and three studies did not consider food-products), one entailed a correction on an article that was included, six articles that focused on astringency were excluded as they have already been discussed further in two recent review papers on tribology–astringency relationship [20,21], and five papers looked only at oral coatings (deposits on the oral surfaces), not tribology. Once we identified the relevant studies, we decided to focus on the 16 most recent papers (published in or after 2016). Relevant information from these 16 selected studies, such as details on the tribology measurements and sensory test method, were extracted and are reported in Table 1.

**Table 1:** Food science studies that have examined tribology–sensory relationships

Lubricant	Tribology	Simulated oral conditions	Sensory	Statistical analysis	Correlation	References
o/w emulsions	Lab-modified TA (0.1–40 mm/s, 0.57 N, PDMS surface and steel balls)	Artificial saliva (ions, mucin, α-amylase), 28°C	Sensory ratings: compare to reference, *n* = 25	Pearson’s correlations	Smooth (−)	[2^•^]

Microbubble dispersions, o/w emulsions and protein solutions (without/with thickeners or gelling agents)	OTC (10–80 mm/s, 0.5 N, 16 mm oscillation, flat-bottom PDMS probe and glass surface)	No saliva, 20°C	Tetrad test, *n* = 7			[35]

o/w emulsions and emulsion-filled gels; Emulsion-filled mixed gels, after simulated oral processing	OTC (10–80 mm/s, 0.5 N, 16 mm oscillation, flat-bottom PDMS probe and glass surface)	No saliva, 20°C; 37°C	QDA, *n* = 12	Pearson’s correlations	Sticky [*first bite*], sticky, rough, powdery, spreadable, fatty [*chew down*], fatty, dry [*after-feel*]	[37,36^••^]

Hydrogels, after simulated oral processing	MTM2 (1–1000 mm/s, 2 N, PDMS ball-on-disc set-up)	Artificial saliva (ions, mucin), 37°C	Descriptive analysis, *n* = 11	Pearson’s correlations	Pasty (−), slippery, salivating	[1^••^]

Milk (0.2–3.25%; 0.2–5% fat)	Tribo-rheocell accessory (0.15–750 mm/s, 1 N, double-polypropylene ball on PDMS disc)	Human saliva (stimulated); no saliva, 25°C	Paired comparison (2-AFC), *n* = 24; Spectrum descriptive analysis, *n* = 7	Regression analysis	Astringency	[24,29^•^]

Yoghurt, soft cream cheese (low–high fat)	MTM (1–1000 mm/s, 2 N, PDMS ball-on-disc set-up)	Artificial saliva (ions, mucin), 37°C	Triangle test and intensity scoring, *n* = 63			[6^•^]

Yoghurts (0% milk fat) with different casein to WP ratios	Tribo-rheocell accessory (0.001–1000 mm/s, 3 N, stainless steel ball on rubber pads)	No saliva, 10°C	Descriptive analysis, *n* = 7	Pearson’s correlations	Gelatinous, aerated, lumpy, grainy, adhesive (−), creamy (−), smooth (−) [*in-mouth*], difficult to swallow (−), mouth coating (−) [*after-feel*]	[23]

Yoghurts (with added protein and modified starch)	Lab-modified TA (0.1–10 mm/s, 0.27 N, silicone elastomer surface and steel balls)	Human (stimulated) and artificial saliva (ions, mucin, α-amylase), 25°C	Flash profiling, *n* = 13			[38]

Pot-set (0.1–3.8% fat) or stirred yoghurts (0.1% fat), with added thickeners	Tribo-rheocell accessory (0.01–100 s^−1^, 2 N, half-ring on surgical tape plate)	No saliva, 35°C	QDA, *n* = 8	Ranking of products according to the different parameters		[10,39]

Custard dessert formulations	Tribo-rheocell accessory (0.01–6.5 rad/s, 2 N, half-ring on surgical tape plate)	No saliva, 35°C	Ranking descriptive analysis, *n* = 11, *not for all samples			[25]

Cream cheese (different fat content)	Tribo-rheocell accessory (0.1–600 s^−1^, 2 N, ring on surgical tape plate)	No saliva, 35°C	TDS, *n* = 10			[40^•^]

Milk chocolates	Tribo-rheocell accessory (0.02–750 mm/s, 3 N, stainless steel ball on PDMS plates)	Human saliva (stimulated), 40°C	QDA, *n* = 12			[9]

Gluten-free bread (with different modified dietary fibres)	Rheometer with custom-made head (1 mm/s, 0.2 N, three steel balls on bread taped to plate)	No saliva, 20°C	Time-intensity, *n* = 10	Pearson’s correlations	Firm, chewy, dry	[41]

## Oral tribology in food science

### Instruments

**Figure 2:**
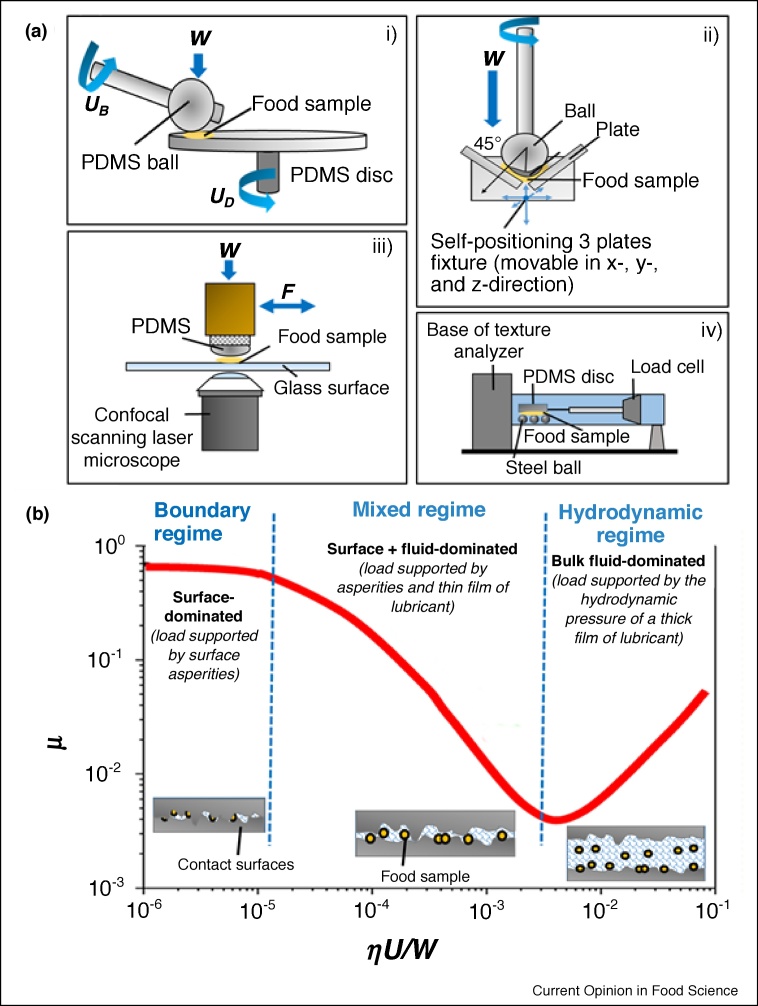
Schematic illustration of different tribometers that are used in the area of food research **(a)** showing a Mini-traction-machine (MTM) with PDMS ball-on-PDMS disc set-up, where *U_B_* and *U_D_* are the speeds of the ball and disc, respectively and *W* is the load, **i**) (*redrawn from* Ref. [6^•^]), a Tribo-rheocell accessory that is a ball-on-three plate set-up as an attachment to a controlled stress rheometer, **ii**) (*redrawn from* Ref. [30]), an optical tribological configuration (OTC), where *F* is the frictional force, **iii**) (*redrawn from* Ref. [31]), a lab-modified texture analyser with steel ball-on-PDMS disc set-up, **iv**) (*redrawn from* Ref. [32]); and a typical Stribeck curve showing the friction coefficient (*μ*) between surfaces as a function of the combined lubrication parameters of the lubricant viscosity (*η*), entrainment speed (*U*) and load (*W*), **(b)** (*redrawn from* Refs. [11^••^,14^•^]).

In recent years, an impressive suite of commercially available and bespoke tribometers have surfaced to quantify the friction in presence of model and real food systems (Figure 2a), and allowed the plotting of Stribeck curve (Figure 2b). The differences between these tribometers are often the range of speed, material properties of the contact surfaces and the nature of movement (i.e. sliding, rolling, reciprocating). Mini-Traction-Machine (MTM2) by PCS Instruments, UK (Figure 2ai) is one of the most commonly used tribometers [1^••^,^4^,^5^,6^•^,^22^] that employs a ball-on-disc set-up with load range varying from 1 to 8 N (low load beam) in a combined sliding/rolling configuration (0–200% slide-to-roll ratio) and features a relatively wide range of entrainment speeds (1 × 10^−3^–3 m s^−1^). To emulate the viscoelastic properties of tongue and oral palate surfaces, the conventional stainless steel tribopairs have been replaced by compliant elastomers, that is polydimethylsiloxane (PDMS) in the recent food science literature. It is noteworthy that the contact pressure even using such PDMS ball-on-discs can be almost an order of magnitude higher than that found in mouth conditions [11^••^].

Besides these tribometers that can be purchased off-the-shelf, tribo-rheo cells have gained popularity among food scientists as they allow attaching an accessory to a controlled-stress rheometer, such as a ball-on-three plate set-up offered by Anton Paar, Austria [9,23] and double ball-on-plate [24] to ring-on-plate geometries [25] from TA Instruments, USA. An example of such a tribo-cell accessory [26,27] is shown in Figure 2aii, where a non-conforming ball-on-three plates contact is mounted on a movable stage, allowing even distribution of the load on all the ball-plate contact points. This tribo-rheo cell allows a wider sliding speed range, particularly in the lower speed region (1 × 10^−5^–1.4 m s^−1^) [28], but only allows sliding motion as compared to the sliding, rolling or reciprocating motions in MTM. The tribopairs used in these tribo-rheo cell experiments have ranged from steel/PDMS [9], polypropylene/PDMS [24,29^•^], steel/rubber [23] to steel/rough surgical tapes [10,25], making it difficult to compare data even if the same food is used as a lubricant. This is because tribology is not only influenced by the lubricant but also by the material properties (surface roughness, viscoelasticity, wettability) of the surfaces in contact, the interactions between such lubricant and the surfaces (i.e. hydrophobic, electrostatic or van der Waals) and the motion of the surfaces (i.e. entrainment speed) unlike rheology, which is a material property of the food [11^••^].

In-house laboratory-made tribometers have also been engineered by few research groups, such as the optical tribometer cell (OTC) with PDMS-glass contact surfaces (Figure 2aiii) [3,31] and the three steel balls-on-PDMS disc set-up in a modified texture analyser (Figure 2aiv) [2^•^,^32^], both working on a limited sliding speed range (1–8 × 10^−2^ m s^−1^ or 1 × 10^−5^–4 × 10^−2^ m s^−1^, respectively). The OTC has an advantage of real-time visualization of the sample during tribological stress using a confocal microscopy; however, it has the limitation of one surface always being glass to allow the visualization.

Besides the geometry, researchers have recently started to emulate the movements of the tongue against the palate in the tribological set-ups and examined its impact on friction force. For instance, yoghurts with different fat contents showed significant differences in the friction coefficients (*μ*) values by using sliding-reciprocating motion with restricted stroke length (<contact width) [33]. Particularly, the replenishment of yoghurt was controlled by periodic loading and unloading of the reciprocating motion similar to what is expected to happen in real-mouth conditions. It can be deduced that oral friction is time-dependent rather than shear/entrainment speed-dependent. In another case with oil-in-water emulsions, the dependence of *μ* on oil content in emulsions changed significantly when the motion was changed from reciprocal linear to semi-elliptical movements [34]. To mimic the loads in real oral environment, the nanotribometer has been introduced in food science (CSM, Switzerland) [7], which can employ two orders of magnitude lower normal force than the aforementioned tribological set-ups. However, considering the modulus of the polymeric surfaces used in this study [7], even at 30 mN load range, the pressure was still comparable to that obtained in MTM2 machines [11^••^].

### Lubrication regimes

The output from these afore-mentioned tribometers is generally plotted in the form of a curve where *μ* varies as a function of the entrainment speed of the lubricant. When the abscissa is a combined factor of lubrication parameters, that is, contact load (W), the lubricant viscosity (*η*) and the lubricant entrainment speed (*U*), it is referred to as Stribeck curve [11^••^] (Figure 2b). In case of oral tribology, lubricant generally means saliva, food or a food–saliva mixture. As the Stribeck curve progresses, three different lubrication regimes can be identified: the boundary, mixed and hydrodynamic regimes. When the speed of relative motion of the contacting surfaces (tongue-palate contacts or instrumental ball-disc contacts) is small, there is not enough lubricant that enters or stays in between the two surfaces. This is known as the ‘boundary regime’, where the properties of the contacting surfaces, such as surface roughness, dominate the friction behaviour rather than the viscosity of the lubricant. For instance, extension of the boundary regime toward higher speeds might suggest rough papillae-dense surfaces of the tongue plausibly interlocking with the palate in absence of a continuous lubricating film. Such asperity interlocking might result in high *μ* values and can be the physical mechanism behind mouthfeel perceptions of ‘roughness’ or ‘dryness’. The magnitude of *μ* thus provides useful information about the surface chemistry of both the contact surfaces as well as the thin lubrication film that may be a few molecules thick in the boundary regime.

As the entrainment speed increases (Figure 2b), more lubricant starts entering the contact region allowing better surface separation. Here, the lubricant forms a thin continuous film capable of partially supporting the load, consequently decreasing *μ* values, known as the ‘mixed regime’. Here, not only the material properties of both the lubricant and the surfaces, but also the interactions between the lubricant and surfaces are of great importance in determining the *μ* value. Thus, the onset and continuity of this regime are mostly useful for understanding the lubrication behaviour of foods. As the entrainment speed increases further, the ‘hydrodynamic regime’ is reached, where a thick film of lubricant totally separates the surfaces by sustaining the applied load. Here, the bulk properties (i.e. viscosity, structure) of the lubricant dominate the friction behaviour.

## Sensory tests used in oral tribology domain

A wide range of standard sensory tests have been used by oral tribology scientists, where number of participants have ranged from 7 to 63 (Table 1). Sensory discrimination tests, such as the two-alternative forced choice (2-AFC) paired-comparison test, triangle test and tetrad test have proven to be useful to discriminate dairy products differing in ‘creamy’ [6^•^] or ‘astringency’ mouthfeel [24]. Although these sensory methods do not allow for quantitative correlations of different attributes with tribology, difference/increase in *μ* might be the main reason for the fact that most panellists could distinguish these products. For instance, an elegant study [35] using tetrad tests allowed the conclusion that microbubbles are not suitable for direct fat replacement as panellists differentiated model food systems containing emulsion droplets from those containing microbubbles of similar size (1 μm). This can be qualitatively related to higher *μ* values in the microbubble-containing system in absence of droplet coalescence-induced oil film formation in the contact surface. Such film forms in the emulsion droplet containing counterpart and reduces *μ*. Also in real foods, such as yoghurts, triangle tests with untrained panellists (*n* = 63) [6^•^] has allowed sensory discrimination of iso-viscous commercial yoghurts with different fat content. Although rheology could not discriminate these products, *μ* values in the boundary and mixed regimes were one-order of magnitude lower for full-fat yoghurt as compared to the no-fat counterparts, giving qualitative indications about relationships between sensory perception and tribology.

Indeed, descriptive sensory techniques, such as Quantitative Descriptive Analysis™ (QDA) and Spectrum™, have been preferred by various research groups (Table 1). Such techniques require a relatively small number of trained panellists and allow examining quantitative relationships of specific attributes with *μ* at a particular speed [1^••^,^10^,29^•^,36^••^]. However, descriptive sensory techniques require extensive training of the panellists, and consequently are time-consuming and expensive. In addition, maintaining such a trained panel and finding appropriate standards for training on friction-related sensory attributes can be challenging. Moreover, testing with unfamiliar model foods, such as gels, might require additional hours of training. In such cases, it is crucial to check panel performance that is agreement, discrimination and repeatability among panellists, to be statistically acceptable before using such data for correlations with tribology [1^••^].

## Correlating *μ* and sensory attributes

Quantitative relationships between *μ* values at a particular speeds and specific sensory attributes evaluated by panellists have attracted significant research attention in both model foods (emulsions, emulsion gels and hydrogels) and real foods (milk, yoghurts, custards, cream cheese, chocolate and bread) (Table 1). For instance, in whey protein-stabilized emulsion-artificial saliva mixtures [2^•^], Pearson’s correlation showed that *μ* in the mixed regime (15–30 mm/s) correlated inversely with sensory ‘smoothness’ (*R*^2^ = 0.95–0.98, *p < 0.005*). Also, in simulated boli of emulsions gels (agar/gelatine-based) [36^••^], friction force in the mixed regime (80 mm/s) correlated directly with sensory ‘stickiness’ (*R*^2^ = 0.59–0.76, *p < 0.05*). In contrast, the only study that has looked at tribology–sensory relationships in aqueous hydrogels [1^••^] suggests that the *μ* of the hydrogel bolus filtrate (i.e. gel particles >500 μm were filtered out, after simulated oral processing in presence of artificial saliva) in the mixed regime (50 mm/s) correlated inversely with sensory ‘pastiness’ (*R*^2^ = 0.80, *p < 0.05*) and positively with ‘slipperiness’ (*R*^2^ = 0.82, *p < 0.05*), as well as ‘salivating’ (*R*^2^ = 0.79, *p < 0.05*). The sign of correlations of *μ* with sensory attributes in hydrogels might be surprising if compared against those with fat-based emulsion gels. Noteworthy, ‘pastiness’ in this hydrogel [1^••^] was postulated to be associated with the mouth-coating aspects of hydrogel bolus filtrates that is the coating was viscous enough to separate the oral surfaces and thus reduction in *μ* was observed in the ‘pasty’ samples. In contrast, the ‘slippery’ perception was defined by the ease of sliding meaning that highly slippery gel boli was easily sliding past the oral surfaces. This led to the gel boli not being retained within the contact surfaces, resulting in high *μ* values [1^••^]. Mechanistically, these three studies on model systems suggest that certain surface-related sensory attributes can be mathematically expressed as a *f*(*μ*), where the derivative can be either increasing or decreasing. Also, the relationship with *μ* is often at a specific speed within the mixed regime, that is at speeds within the range of real tongue movements [11^••^]. Noteworthy, the use of artificial saliva in tribology experiments improved the strength of the relationship with sensory attributes [1^••^,2^•^], a role which is often ignored (Table 1).

Tribology of real food samples is not envisaged to replace sensory analysis in food industries, but it enables building physically relevant hypotheses behind perception and thus helps to navigate product development in the right direction. Furthermore, tribology can be used for textural pre-screening and selection of a small set of optimized samples for panel testing, thus reducing product development time and costs. In real food systems (Table 1), *μ* has thus been mainly correlated with fat-related attributes (e.g. smoothness, creaminess) in semi-solid dairy products (typically yoghurt) by comparing full-fat products with those containing fat mimetics, such as starch and non-starch polysaccharides [6^•^,^10^,^25^,^39^]. More recently, the focus has shifted toward non-fat related perceptions. For example, *μ* in the mixed regime (100 mm/s) correlated positively with sensory astringency in milks (*R*^2^ = 0.71–0.74), giving indications of heat-induced protein aggregation that influenced both instrumental friction profiles and astringency perceptions among trained panellists [29^•^]. Besides liquid and semi-solid foods, tribology in solid foods has also been attempted. In a recent study using steel and gluten-free bread samples as counter surfaces, *μ* was positively correlated with the perceived firmness (*r* = 0.90, *p < 0.05*), chewiness (*R*^2^ = 0.85, *p < 0.05*) and dryness (*R*^2^ = 0.94, *p < 0.05*). However, the regime in which such correlations exist is not clear. These empirical oral tribology–sensory correlations in real foods are providing useful evidence for estimating the potential of tribology to predict the sensory perception beyond conventional fat-related attributes. However, in-depth physical causalities behind such relationships are often difficult to interpret owing to the complexity of food structure.

**Figure 3:**
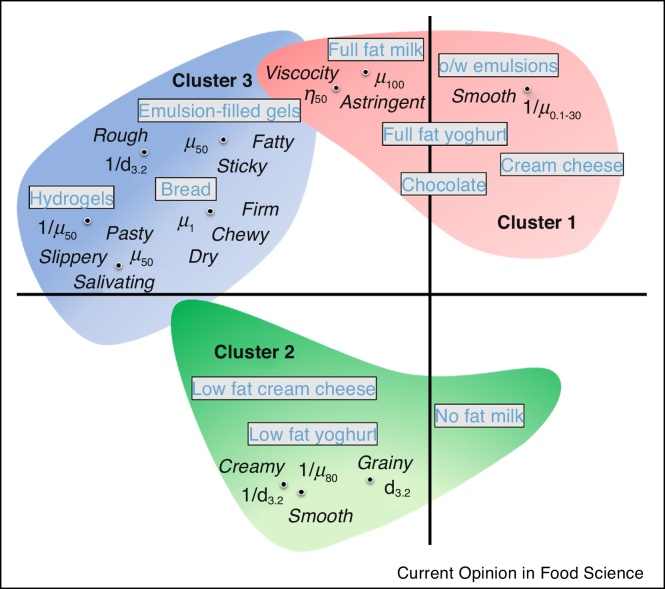
Schematic representation of qualitative clusters on correlations between instrumental and sensory parameters for different model and real products, based on studies reported in Table 1. Here *μ*, *η* and *d*_32_ represent the friction coefficient, viscosity and mean particle size, respectively. The subscripts for *μ* and *η* are the speed (mm/s) and shear rate (s^−1^), respectively.

In Figure 3, we have plotted a schematic representation summarizing possible existing correlations in different test foods (both model and real food systems) between lubrication-related sensory attributes as well as other relevant instrumental parameters, such as friction coefficients, viscosity and particle size, based on Table 1. As can be seen (Figure 3), three clusters were identified: 1) foods containing fat, 2) no-fat to low-fat containing foods, and 3) a variety of different solid model and real foods. For clusters 1 and 2, the relevant sensory attributes were smooth, creamy, viscous, astringent and grainy in the fat-related foods, whereas for the solid foods different descriptors were used. The exception being the fat-containing emulsion-filled gels, which showed some overlap with attributes found mainly in cluster 1.

Besides *μ*, many if not most studies in Table 1 have also conducted bulk rheology and particle size analysis that have enabled better understanding of the physical reasoning behind sensory attributes. For instance, besides *μ* in boundary to mixed regimes (30–100 mm/s), sensory viscosity in milks have been found to correlate strongly with instrumental viscosity (*η*) at 50 s^−1^ shear rate [29^•^] (Figure 3). In another case, mean particle size (*d*_3.2_) has been an important factor in understanding the reasoning behind higher *μ* values and corresponding increased sensory roughness [37]. Increase in roughness perception was attributed to the bigger d_3.2_ values, which were much above the sensory detection threshold.

## Closing remarks and future directions

We are at the cusp of a new era in oral tribology where food scientists have already moved on from using steel-steel to steel-PDMS or PDMS–PDMS surfaces to mimic tongue-palate contacts, which have enabled developing empirical relationships with some sensory attributes that are either fat-related or non-fat related. However, considering lubrication is a system property [15^•^] and not an intrinsic material property of the lubricant, generalizability of such relationship can be questioned. It is highly likely that such existing relationship is only valid within the remits of those specific experimental conditions, and such tribology–sensory relationship might not hold well with other equipment or experimental conditions. Hence, it is crucial to build mechanistic hypotheses before trying to examine tribology–sensory relationships. Finally, to marry oral tribology to sensory, the following challenges and future opportunities have been identified:

**Tribometers**. The diversity of equipment makes it difficult to compare *μ* across speeds between different studies. The physics and chemistry of the contact surfaces, as well as the conditions of tribological experiments, such as the load, type of motion and temperature, add significant variance in lubrication performance across laboratories and consequently conclusions related to sensory perception. Therefore, direct comparison of *μ* values across studies must be approached with caution and cross-laboratory studies on same samples should be performed. Of course, development of contact surfaces that emulate real oral surfaces [11^••^] and harmonizing the use of such surfaces across laboratories are still the key unresolved research challenges before the potential of tribology in the food science community can be fully realized.

**Saliva incorporation**. Saliva plays an important role in the oral lubrication processes. Yet, most oral tribology experiments do not incorporate real or artificial saliva, which is particularly important when friction results are correlated with sensory perception. Undeniably, the use of simulated saliva is not the same as using real human saliva, but, incorporation of such saliva containing ions, mucins and amylases [1^••^,2^•^,^4^,6^•^,^42^,^43^] can provide systematic understanding of tribological mechanisms and the perception of complex sensory attributes. It is noteworthy that the human mouth is hydrophilic due to the coating of saliva on otherwise hydrophobic mucosa [11^••^]. Hence, experiments using both hydrophobic as well as saliva-coated hydrophilic polymeric substrates [5,44] are recommended to elucidate the role of surface chemistry-driven phenomena behind tribology–sensory relationships.

**Rheology and microstructural analysis**. Many, if not most, oral tribology studies considered in this paper have included apparent viscosity measurements that are particularly important to understand the tribology results in the hydrodynamic lubrication regime. We recommend also supporting such experiments with particle sizing and microscopy that can be beneficial to understand the underlying mechanism behind tribology–sensory correlations.

**Sensory science**. Although conventional descriptive tests have allowed elegant progress, novel sensory methods such temporal testing [40^•^,^41^,^45^], which determine dynamic textural perceptions can bring new perspectives on time-dependent correlations with friction parameters.

**Statistical analyses**. Finally, most of the sensory attributes, such as creaminess and astringency, are multimodal sensations. Thus, besides Pearson’s correlations, multivariate analysis, such as Principal Component Analysis (PCA), Partial Least Squares Regression (PLS) and pattern recognition incorporating multiple physical parameters need to be investigated. This might be beneficial to understand the exact contribution of friction to these complex sensory attributes and allow the development of tribology-based predictive equations for specific sensory perceptions.

## Conflict of interest statement

Nothing declared.

## References and recommended reading

Papers of particular interest, published within the period of review, have been highlighted as:

• of special interest
•• of outstanding interest
